# Effect of Nora virus infection on native gut bacterial communities of *Drosophila melanogaster*

**DOI:** 10.3934/microbiol.2021014

**Published:** 2021-06-10

**Authors:** Makayla Schissel, Rebecca Best, Shelby Liesemeyer, Yuan-De Tan, Darby J. Carlson, Julie J. Shaffer, Nagavardhini Avuthu, Chittibabu Guda, Kimberly A. Carlson

**Affiliations:** 1 Biology Department, University of Nebraska at Kearney, 2401 11^th^ Ave, Kearney, NE 68849, USA; 2 Department of Genetics, Cell Biology and Anatomy, University of Nebraska Medical Center, 985805 Nebraska Medical Center, Omaha, NE 68198-5805, USA

**Keywords:** Nora virus, *Drosophila melanogaster*, axenic, gut microbiota, longevity

## Abstract

Gastrointestinal microflora is a key component in the maintenance of health and longevity across many species. In humans and mice, nonpathogenic viruses present in the gastrointestinal tract enhance the effects of the native bacterial microbiota. However, it is unclear whether nonpathogenic gastrointestinal viruses, such as Nora virus that infects *Drosophila melanogaster*, lead to similar observations. Longevity analysis of Nora virus infected (NV+) and uninfected (NV−) *D. melanogaster* in relationship to presence (B+) or absence (B-) of the native gut bacteria using four different treatment groups, NV+/B+, NV+/B−, NV−/B+, and NV−/B−, was conducted. Data from the longevity results were tested via Kaplan-Meier analysis and demonstrated that Nora virus can be detrimental to the longevity of the organism, whereas bacterial presence is beneficial. These data led to the hypothesis that gastrointestinal bacterial composition varies from NV+ to NV− flies. To test this, NV+ and NV− virgin female flies were collected and aged for 4 days. Surface sterilization followed by dissections of the fat body and the gastrointestinal tract, divided into crop (foregut), midgut, and hindgut, were performed. Ribosomal 16S DNA samples were sequenced to determine the bacterial communities that comprise the microflora in the gastrointestinal tract of NV+ and NV− *D. melanogaster*. When analyzing operational taxonomic units (OTUs), the data demonstrate that the NV+ samples consist of more OTUs than NV− samples. The NV+ samples were both more rich and diverse in OTUs compared to NV−. When comparing whole body samples to specific organs and organ sections, the whole fly was more diverse in OTUs, whereas the crop was the most rich. These novel data are pertinent in describing where Nora virus infection may be occurring within the gastrointestinal tract, as well as continuing discussion between the relationship of persistent viral and bacterial interaction.

## 1. Introduction

All living organisms contain microorganisms, such as bacteria and viruses, which are necessary to maintain the health not only of specific organs, but of the host itself. The microorganisms that permanently inhabit the living organism under normal circumstances is referred to as the microbiota or microflora [1]. In order for the microbiota to continue to inhabit the organism, there must be immune tolerance and defense for regulation, which controls it from causing extreme health issues [2]. This type of relationship is difficult to study in vertebrates due to the intricacy of their immune systems and physiology. Therefore, model organisms, such as invertebrates, are essential to studying this complex interaction.

*Drosophila melanogaster*, the fruit fly, is an ideal model organism for laboratory studies that encompass all aspects of immunity, including viral and microbial infection. Advantageous characteristics of *D. melanogaster* include a short life cycle, high reproductive rate, and a relatively small genome. The genome of *D. melanogaster* shows homology with approximately 77% of disease-related genes in humans, which display phenotypes associated with certain immune diseases, such as Severe Combined Immunodeficiency (SCID) and Type-1 Diabetes [3]. Due to the similarity of the genome between *D. melanogaster* and humans, much of what is known about immunity mechanisms against viral and bacterial infections in humans were first discovered in this organism.

Most bacterial species found in laboratory-reared *D. melanogaster* belong to the families *Acetobacteraceae*, *Lactobacillus*, and *Enterobacteriaceae*. However, these bacteria are host-specific and exhibit varying effects in hosts, such as altering metabolism, initiating stress resistance mechanisms, and modifying gut structure [4]–[6]. Nonpathogenic bacteria within *D. melanogaster* enhance longevity [7] and are crucial in promoting a steady rate of the development of larvae and supporting the transition into adulthood [8]. Exposure of commensal bacteria in *D. melanogaster* is most important while the larval fat body is developing into the adult fat body within the pupae [7]. Nonpathogenic bacteria in *D. melanogaster* also work to stimulate host immune responses to pathogenic bacteria [9]. This system works due to the ability of native gut microbes to detect pathogenic bacteria and communicate to the fat body where an immune response should be triggered [10],[11]. After pathogenic bacterial infection is controlled, the nonpathogenic bacteria renew gut epithelium via the Janus Kinase/Signal Transducer and Activator of Transcription (JAK-STAT) pathway. When bacteria are not present due to axenic conditions and/or the downregulation of the JAK-STAT pathway, gastrointestinal epithelial renewal is severely affected [12]. In addition to the roles of nonpathogenic bacteria and immunity, these innate bacteria also have the ability to influence mating patterns. When native bacteria are not present within the gastrointestinal tract of *D. melanogaster*, fecundity and desire to mate are negatively impacted. This is most likely due to the organism's increased energy expenditure in sustaining life [13]. A role of the native bacterial microbiota is to maintain gut health, which includes preventing viral infection. For example, the commensal bacteria located within the gut of mosquitoes has demonstrated the ability to halt Dengue virus replication via the activation of immunity genes regulated by Toll-like receptor (TLR) [14]–[17]. In *D. melanogaster*, Drosophila C virus, an enteric virus, induced the gut microbiota to prime antiviral signaling to restrict infection [18]. Similar effects may result from the presence of viruses, but this is largely unaddressed in *D. melanogaster*.

In *Drosophila*, the gut and the fat body constitute the primary immune tissues. Whereas the gut is responsible for viral replication and absorption of nutrients, the fat body is important for nutrient storage and being the primary site of antimicrobial peptide (AMP) production. When exogenous bacteria were added to the diet at day 0 of adult life, this extended female survival through modulation of nutrient reserves by the fat body [19]. In the brown planthopper (BPH), the microbiome of the fat body was characterized in response to antimicrobial treatment. The results demonstrated that the microbes present in the fat body play a critical role in the BPH life cycle [20]. Interestingly, the microbiome of the fat body in *D. melanogaster* is largely unstudied under any condition.

Due to the location and abundance of bacteria in the gastrointestinal tract and the role of the fat body in AMP production, it is hypothesized that viruses interact with the native bacteria in these areas and affect health and longevity. Nonpathogenic viruses present in mice, humans, and *D. melanogaster*, have been hypothesized to have gut restoration capabilities following antibiotic usage or other axenic treatment [21],[22]. Nora virus is a picorna-like virus that is commonly found in *D. melanogaster* laboratory stocks. Prevalence in wild populations is unknown but is predicted to be 5–32% [23]. Like other viruses belonging to the *Picornaviridae* superfamily, Nora virus is a single-stranded, positive sense RNA virus. However, it has a larger genome (~12 kb), four open reading frames (ORFs), and a unique capsid structure. This suggests Nora virus should be recategorized as a different family [24],[25]. Nora virus is transmitted through the fecal-oral route and replicates in the gastrointestinal system of *D. melanogaster* [26]. Unlike other viruses that persist in *D. melanogaster* populations, Nora virus leads to a chronic infection without pathogenicity [26] but negatively affects geotaxis/locomotor ability [27]. The interaction between Nora virus and native bacteria has not been investigated. However, data show that gastrointestinal viruses, such as poliovirus and human/murine norovirus, can utilize commensal bacteria in the gut to replicate. This allows viral load to increase and bacterial load to decrease during viral infection [28]. One objective of this study was to determine whether there was a difference in longevity between *D. melanogaster* infected with Nora virus (NV) or left uninfected, and the interaction of these conditions with the native bacteria being present or absent. Results from these experiments led to the exploration of gastrointestinal and fat body bacterial composition between NV+ and NV− *D. melanogaster*. Currently, the literature is limited when considering the interaction of virus and innate gut bacteria in *D. melanogaster*, and information on specific organs or organ sections, such as the fat body, has not been characterized. It was hypothesized that Nora virus infected and uninfected flies would contain varying compositions of bacterial microbiota housed within the gut and the fat body. In addition, this interaction was determined for the sections of the gut, the fat body, and whole flies, which has not been done before when considering the interaction between a virus and the microbiota.

## 2. Materials and method

### 2.1. D. melanogaster husbandry and Nora virus infection

Witi *Rel^E23^ D. melanogaster* stocks, a kind gift from Dr. Dan Hultmark, were used for Nora virus infection [29] and the creation of axenic flies. This stock is a precision P element excision line that retains wild-type function and is used as a control for the other *Relish* mutant stocks generated at the same time [30]. It has been used in other published studies characterizing Nora virus [23],[24],[27]. Stocks were maintained on standard cornmeal-molasses-yeast food at ~25 °C with diurnal light. For the infected stocks, one hundred Nora virus infected *D. melanogaster* males were placed in each of 6 bottles with standard cornmeal-molasses-yeast food. Six replicate bottles with one hundred Witi *Rel^E23^* uninfected males served as the control. The males were allowed to defecate on the food for 6 days to ensure adequate transfer of Nora virus to the surface of the food in the experimental bottles. After six days, the males were removed and killed, and 25 female and 25 male Witi *Rel^E23^* uninfected flies were added to each of the 6 Nora virus infected and Witi *Rel^E23^* uninfected bottles. The flies were allowed to mate, lay eggs for 5 days, and morgued. The F1 generation was allowed to emerge and age for approximately 4 days to ensure productive infection before generation of the axenic treatment groups. Ten bottles of each set of conditions housed the following treatment groups: Nora virus positive/bacteria positive (NV+/B+), Nora virus negative/bacteria positive (NV−/B+), Nora virus positive/bacteria negative (NV+/B−), and Nora virus negative/bacteria negative (NV−/B−). Stocks were expanded approximately every 4–5 days. The four different treatment groups were maintained in four separate incubators and dissected in separate rooms to prevent cross-contamination.

### 2.2. Axenic media

Standard cornmeal-molasses-yeast food was prepared, autoclaved for 30 min, and cooled to 55 °C. All bottles, flasks, beakers, and other instruments were autoclaved for 15 min and irradiated under UV light for 1–2 h prior to use. The sterile axenic food bottles contained antibiotic-infused food by adding 2 mL of a 100X stock of antibiotics, consisting of 500 µg/mL ampicillin, 50 µg/mL tetracycline, and 200 µg/mL rifamycin, in 50% ethanol per 100 mL of liquefied food. For the control, non-antibiotic food was generated by adding 2 mL of 50% ethanol per 100 mL of liquefied food.

### 2.3. Generating axenic germ-free D. melanogaster cultures

The process of establishing germ-free flies was according to the protocol published by Brummel *et al*. [7]. Using a germ-free laminar flow hood, 24–48 hour old embryos were dechorionated in 2.7% sodium hypochlorite (bleach) for 2 min and washed twice in 70% ethanol to remove surface yeast and bacteria. The sterile embryos were washed twice with sterile, distilled water to remove traces of ethanol and bleach. Within the germ-free hood, the embryos were transferred to a pre-autoclaved and irradiated bottle containing the antibiotic food and capped with a pre-autoclaved and irradiated foam plug. Non-axenic embryos were washed four times in sterile, distilled water and transferred to a pre-autoclaved, irradiated bottle containing non-antibiotic food and capped with a pre-autoclaved and irradiated foam plug.

### 2.4. Confirming axenic germ-free cultures and identification of microbes

To ensure that axenic stocks contained no bacteria, axenic flies were homogenized and plated on LB agar plates with no antibiotics following the protocol published by Brummel *et al*. [7]. The plates were allowed 24–48 hours to incubate at 37 °C. Gram staining and additional observations were performed. Fly stocks confirmed to be bacterial free were transferred to new, autoclaved, and irradiated food bottles every 4–5 days. The axenic food bottles were spot checked for contamination by periodically swabbing the food in the axenic vials, plating the culture on LB plates, and incubating at 37 °C for 24–48 hours.

### 2.5. Confirmation of infection of Nora virus

To ensure the flies had remained Nora virus infected or uninfected, RT-PCR of the *ORF1* region was performed. RNA was extracted from the longest surviving flies of each treatment group using TRIzol^®^ according to manufacturer's instructions (Invitrogen, Carlsbad, CA). *Nora ORF 1* 54–844 (F 5′-TGGTAGTACGCAGGTTGTGGGAAA-3′; R 5′-AAGTGGCATGCTTGGCTTCTCAAC-3′) specific primers and Promega Access Quick RT-PCR master mix were used according to manufacturer's instructions (Madison, WI) to analyze for the presence of Nora virus [29]. Thermocycler conditions were as follows: 50 °C for 30 min, 94 °C for 2 min (94 °C for 30 s, 55 °C for 1 min, 68 °C for 1 min)_30 cycles_, 68 °C for 5 min, and held at 4 °C. A product of ~790 bp was expected in Nora virus infected cultures.

### 2.6. Longevity analysis

Three longevity cages approximately a liter in volume (~ 16 cm in total height) with ventilation and food vial extension tube were used for each of four groups (NV+/B+, NV−/B+, NV+/B−, NV−/B−). The cages were disinfected and irradiated under UV light before introducing flies. Sixty virgin female *D. melanogaster* were collected and placed into each of three cages for each treatment (3 biological replicates for each treatment). Vials of antibiotic or non-antibiotic food were changed every 3–4 days. Beginning at day 1 in the cage, dead flies were removed by aspiration and tallied every three days. Dead flies were frozen at −80 °C for future RNA analysis. Percent mortality and survivorship were calculated using a Kaplan-Meier Survival Curve analysis. These curves were used to compare the total number of deaths over time in each treatment group.

### 2.7. Dissection preparation and extraction

*D. melanogaster* from the four treatment groups were reared on sterile, autoclaved food. Virgin females were collected every 6 hours and placed on new autoclaved food in order to age for 4 days. Flies were anesthetized and placed in a 10% sodium hypochlorite solution for 10 min to remove any exterior bacteria, followed by a *Drosophila* Ringer's solution rinse. The gastrointestinal tract was dissected under sterile conditions, using a laminar flow hood, and separated into three main parts, the crop (foregut), midgut, and hindgut. The fat body was also collected. Dissections were conducted in triplicate (~30 flies per replicate) using a basic dissecting light microscope with the use of forceps that were sterilized after each dissection via the Germinator 500™.

### 2.8. DNA extractions and 16S next generation sequencing

DNA was extracted from whole flies, sterilized whole flies, each gut section, and the fat body using a Qiagen DNeasy® Blood & Tissue kit. Extractions were quantified and purity was assessed via Nanodrop™. The University of Nebraska Medical Center (UNMC) Genomics Core facility required a minimum concentration of 15 ng/µL of DNA to be viable for 16S rRNA sequencing using a next generation sequencer (NGS), NextSeq 500. Quantified samples were sent to UNMC for 16S sequencing, where all DNA samples were amplified using primers for the V4 hypervariable region of the 16S rRNA gene and DNA sequencing was performed using Illumina's NGS technology.

### 2.9. Metagenome analysis

The 16S rRNA gene sequencing data obtained from multiple body sites of the *D. melanogaster*, the crop, midgut, hindgut, fat body, whole fly with and without Nora virus infection in triplicate were analyzed using QIIME2 version 2019.4 suite [31]. The paired-end 16S sequencing data (in Casava 1.8 demultiplexed fastq format) obtained from all thirty-six samples were imported into QIIME2 environment as QIIME2 artifacts (.qza files). The paired-end DNA-seq reads were denoised, dereplicated and quality filtered using q2-dada2 plugin [32] or deblur [33]. The default settings of trim and truncate options in dada2 denoise-paired method were modified based on quality scores of the sequencing reads. The forward reads were trimmed 6 nucleotides from left and truncated to base pair 293 from the right and reverse reads were trimmed 6 nucleotides from left and truncated to base pair 250 from right. The q2-dada2 and q2-deblur pipelines were used to denoise the sequencing reads into amplicon sequence variants (ASVs) and outputs them as a feature table and representative sequences.

The features of the ‘Pseudomonas’ genus was filtered from the feature table based on the taxonomic labels using q2-taxa plugin. This was due to the overabundance of *Pseudomonas* in the initial analysis that is an established contaminant in commercial solutions. ASVs (as representative sequences) were assigned with taxonomic labels using q2-feature-classifier plugin implemented with pre-trained Naïve Bayes classifier, trained on ‘Greengenes 13_8 99% OTUs from the 515F/806R region of sequences’. The feature table together with an ASVs taxonomy table and sample metadata were converted into an abundance table and viewed as taxa bar plots using a qiime2 taxa barplot plugin. Relative abundance of major taxa at phylum, order and species taxonomic levels was represented as bar plots to show taxonomic diversity among the samples. Microbial community diversity analysis was performed based on rooted phylogenetic tree, generated by multiple alignment of representative sequences using mafft-fasttree method implemented in q2-phylogeny. The diversity analytic plugin, q2-diversity was used to calculate multiple alpha and beta diversity metrics. Shannon metrics [34], evenness index [35],[36], observed OTUs, and Faith-phylogenetic metrics (Faith-pd) [37] were used to evaluate alpha diversity in body sites and sub-body sites. We used a Kruskal Wallis *H*-test to test for difference between two body sites, two sub-body sites, or multiple body sites with p-value < 0.05. For beta-group diversity analysis, we calculated the UniFrac distance [38] to compare pairwise dissimilarity of samples and used permutation approach to calculate p-values.

## 3. Results

### 3.1. Confirmation of Nora virus and bacterial infection

**Figure 1.**
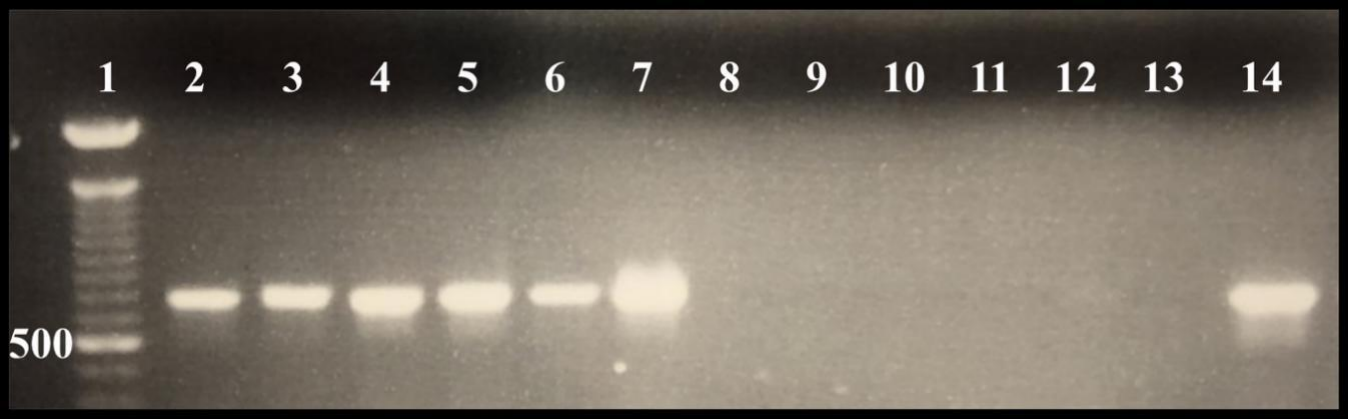
RT-PCR verification of Nora virus infection. Lane 1: 100 bp ladder, Lanes 2–7: Nora virus infected *D. melanogaster*, Lanes 8–12: Nora virus uninfected *D. melanogaster*, Lane 13: Negative control, Lane 14: Positive control. Presence of Nora virus infection was confirmed by an RT-PCR product at approximately 790 bp (lanes 2–7), whereas no PCR product demonstrates no infection (lanes 8–12).

Presence of Nora virus infection was confirmed using Nora virus *ORF1* gene-specific primers and RT-PCR at the end of the experiment using the longest-lived samples for each treatment group. All Nora virus infected stocks were confirmed positive for the virus as demonstrated by a RT-PCR product at approximately 790 bp. All Nora virus uninfected (control) stocks did not demonstrate a 790 bp product (Figure 1). Stocks were expanded from the previously tested bottles and used throughout the entirety of the experiment. Once Nora virus infection status was established, all stocks were tested for the presence or absence of bacteria (data not shown). The stocks cultured on food without antibiotics were expanded and transferred to antibiotic-containing media. The F1 emergents and subsequent generations cultured on antibiotic containing media were found to be axenic, not antibiotic resistant, and were therefore used to execute the longevity analyses.

### 3.2. Longevity analysis

**Figure 2.**
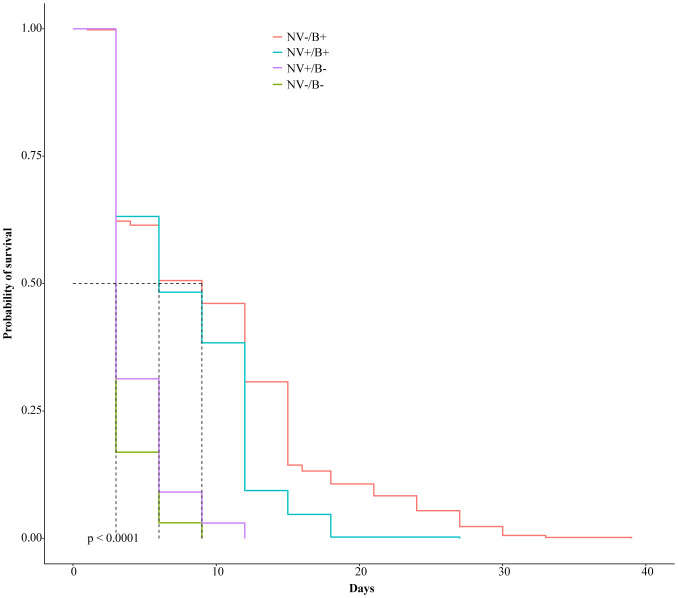
Longevity analysis comparing NV+/− and B+/− treatments. Kaplan-Meyer survivorship analysis demonstrated flies colonized with bacteria lived longer than those without bacteria. Nora virus negatively impacted the life span of flies colonized with bacteria (NV+/B+ vs NV−/B+), but showed an increase in life span of flies not colonized with bacteria (NV+/B− vs NV−/B−). All treatment groups demonstrated significant differences based off a measurement at the 50% survivorship mark and p < 0.0001.

Each cage was monitored for mortality, dead flies collected by aspiration, and data recorded daily. Kaplan-Meier survivorship curves were constructed using the longevity data. Survivorship analysis was done by performing Cox proportional hazards regression. Survivorship analysis showed a significant difference among all four treatment groups (p < 0.0001). In fact, the treatment groups without bacterial colonization died very rapidly compared to the infected groups. Interestingly, the NV+/B− group lived 3 days longer than the NV-/B- group (Figure 2). When Nora virus infection was considered as the variable, the infected group died significantly faster than the uninfected group possessing bacteria (p < 0.0001). When bacterial presence was the variable, the stocks colonized with bacteria lived significantly longer than those without (p < 0.0001 ).

### 3.3. Metagenome analysis

**Figure 3.**
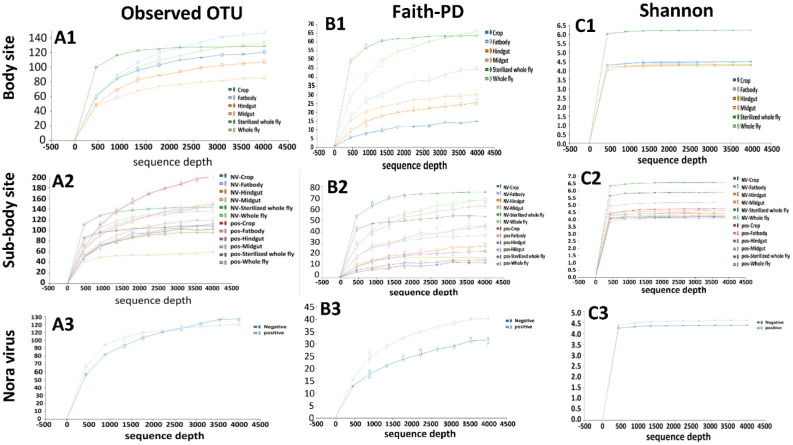
Rarefaction curves for alpha diversity measures of observed OTUs (A1, A2, A3), Faith-PD (B1, B2, B3) and Shannon index (C1, C2, C3) of microbiome of *D. melanogaster* body sites, sub-body sites and negative and positive Nora virus whole fly samples. Faith-PD, the Faith's phylogenetic diversity; Shannon, Shannon-Weiner index; Body sites: Crop, Midgut, Hindgut, Fat body, and whole fly; Sub-body sites: NV-Crop, NV-Midgut, NV-Hindgut, NV-Fat body, and NV-whole fly, pos-Crop, pos-Midgut, pos-Hindgut, pos-Fat body, and pos-whole fly; NV−, negative Nora virus; pos, positive Nora virus.

We performed 16S rRNA V4 amplicon sequencing to study the microbial composition of Norovirus infected and uninfected samples from *D. melanogaster*- crop, midgut, hindgut, fat body, whole fly, and sterilized whole fly in biological triplicates. Sequencing data were analyzed with QIIME2 software. Interactive quality score distribution plots of sequencing reads produced from QIIME 2 analysis demonstrated that the sequencing quality scores decreased as we move toward the 3′ side of both forward and reverse reads. The lower quality scores indicate a greater potential for error in assigning the correct bases towards the 3′ of the sequencing reads. These results indicate that as the length of the read increases, the greater the chance of assigning an incorrect base (Supplementary Figure 1A and 1B). In the majority of the 36 samples analyzed, the sequencing reads ranged from 175,000 to 200,000, with the minimum at 50,000 and the maximum at 275,000 (Supplementary Figure 1C). Both the dada2 and deblur methods were consistent with one another, showing frequency of identified amplicon sequence variants (ASVs)/operational taxonomic units (OTUs) in the samples range from 10–10,000 (Supplementary Figure 1D–1G). We performed the 16S rRNA V4 amplicon sequencing to study the microbial composition of the Nora virus infected and uninfected samples from *D. melanogaster* crop, midgut, hindgut, fat body, whole fly and sterilized whole fly in biological triplicates. From the total 36 samples, we obtained 323 amplicon sequence variants (ASVs)/operational taxonomic units (OTUs) when classified with Greengenes 99% OTUs reference database. Among various body sites, the greatest number of OTUs were observed in the fat body, whereas among the sub-body sites, the greatest number of OTUs were observed in the NV+ crop. When comparing NV+ and NV− samples, NV+ samples consisted of more OTUs than NV− samples. The results from diversity (Faith's PD and Shannon) and richness (observed OTUs) between body sites indicate that the whole fly is the most diverse, while the crop body site is the richest. This suggests that the whole fly contains the most diversity in bacteria, but the crop is the richest in a specific type of bacteria. In terms of sub-body sites, the NV+ fat body is the most diverse while the NV− fat body is the richest. Finally, when comparing NV infected and uninfected samples, NV+ samples are both more diverse and richer than NV− samples (Figure 3).

**Figure 4.**
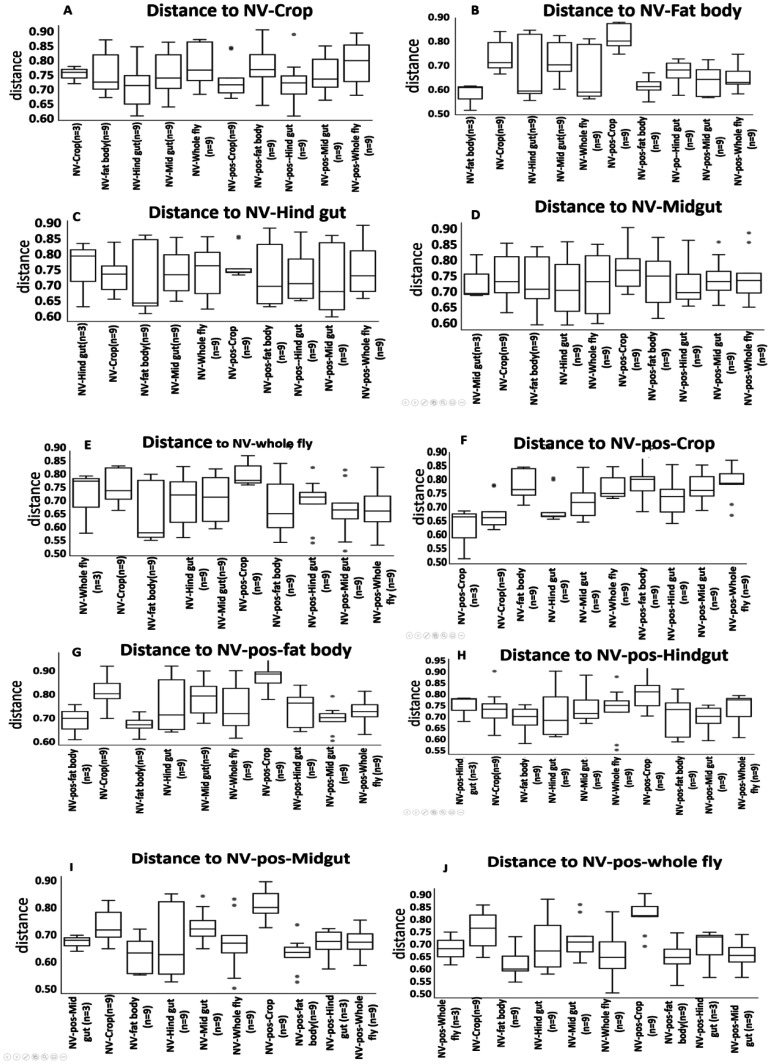
Beta diversity analysis of the microbiome of sub-body sites of *D. melanogaster* with Nora virus infection and no infection using the Catherine Lozupone's weighted Unifrac distance method to measure phylogenetic distance between a sub-body site and the other sub-body sites on the phylogenetic tree. The boxplot displays the phylogenetic distance between a sub-body site and the other sub-body sites. If a sub-body site distances itself in a different phylogenetic trees, the sample size is 3. If sub-body site A distances sub-body site B in different phylogenetic trees, then the sample size is 9. Sub-body sites: NV-Crop, NV-Midgut, NV-Hindgut, NV-Fat body, and NV-whole fly, NV-pos-Crop, NV-pos-Midgut, NV-pos-Hindgut, NV-pos-Fat body, and NV-pos-whole fly; NV−, negative Nora virus; pos, positive Nora virus.

Unifrac distances were measured to assess the variance in bacterial phylogeny between different sub-body sites. Some notable differences include NV- crop and the NV+ crop bacteria had a high phylogenetic distance compared to the NV− fat body bacteria (Figure 4B). The NV+ crop bacteria had a high phylogenetic distance to the NV+ whole fly bacteria (Figure 4E). The NV- fat body bacteria had high phylogenetic distances to NV− crop, NV- hindgut, NV- midgut, and NV+ crop bacteria (Figure 4F). Similarly, the NV+ fat body has high phylogenetic distance to NV− crop, and the NV+ midgut to the NV- crop and NV+ crop is also higher (Figure 4I). The NV− crop and NV+ crop had high phylogenetic distances to NV+ whole fly (Figure 4J). These results suggest that NV− crop and NV+ crop had very different bacteria from NV− fat body, NV− midgut, and the whole fly.

**Figure 5.**
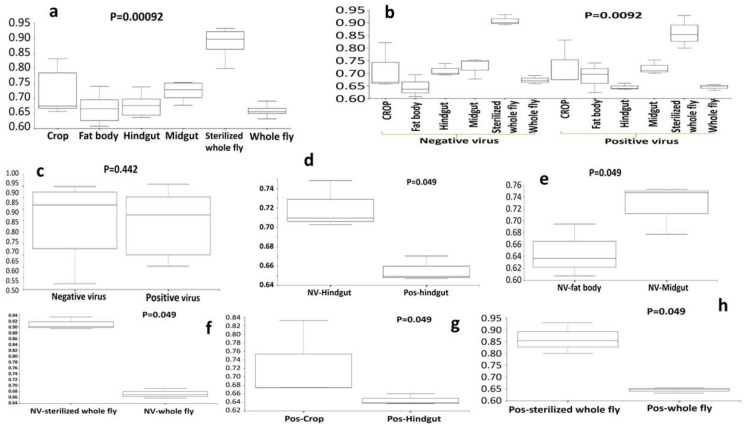
Boxplots of microbial species evenness in different body sites of *D. melanogaster*. Pielou's evenness index of *D. melanogaster* body sites- crop, midgut, hindgut, fat body, whole fly, (a) Negative Nora infection. (b) Negative Nora virus and positive Nora virus infection. (c) Negative and positive Nora virus infected whole fly. (d) between NV-Hindgut and pos-Hindgut. (e) between NV-Fat body and NV-Midgut. (f) between NV-sterilized Whole flies and NV-Whole flies. (g) between pos-Crop and pos-Hindgut. (h) between pos-sterilized Whole flies and pos-Whole flies. NV−, Negative Nora virus infection; pos, Positive Nora virus infection.

**Figure 6.**
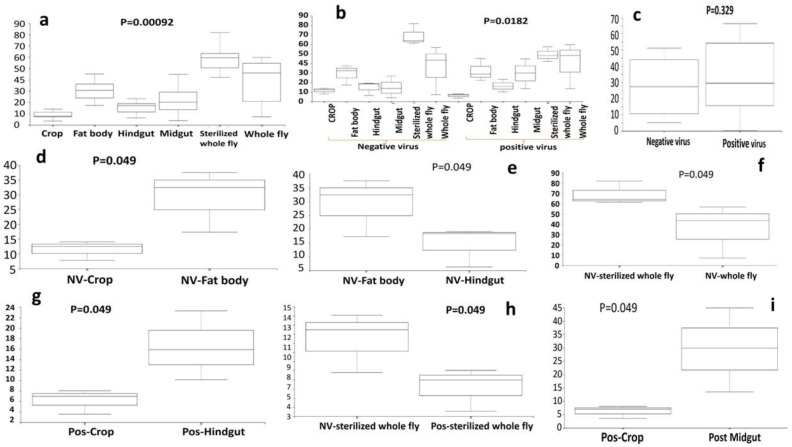
Boxplots of diversity of microbial communities of different body sites of *D. melanogaster*. Faith's phylogenetic diversity of *D. melanogaster* body sites- crop, midgut, hindgut, fat body, sterilized whole fly and, whole fly, (a) Negative Nora infection. (b) Negative Nora virus and positive Nora virus infection. (c) Negative and positive Nora virus infected whole fly. (d) between NV-Crop and NV-Fat body. (e) between NV-Fat body and NV-Hindgut. (f) NV-sterilized whole fly and NV-whole fly. (g) between pos-Crop and pos-Hindgut. (h) between NV-Sterilized whole fly and pos-Sterilized Whole fly. NV−, Negative Nora virus infection; pos, Positive Nora virus infection.

**Figure 7.**
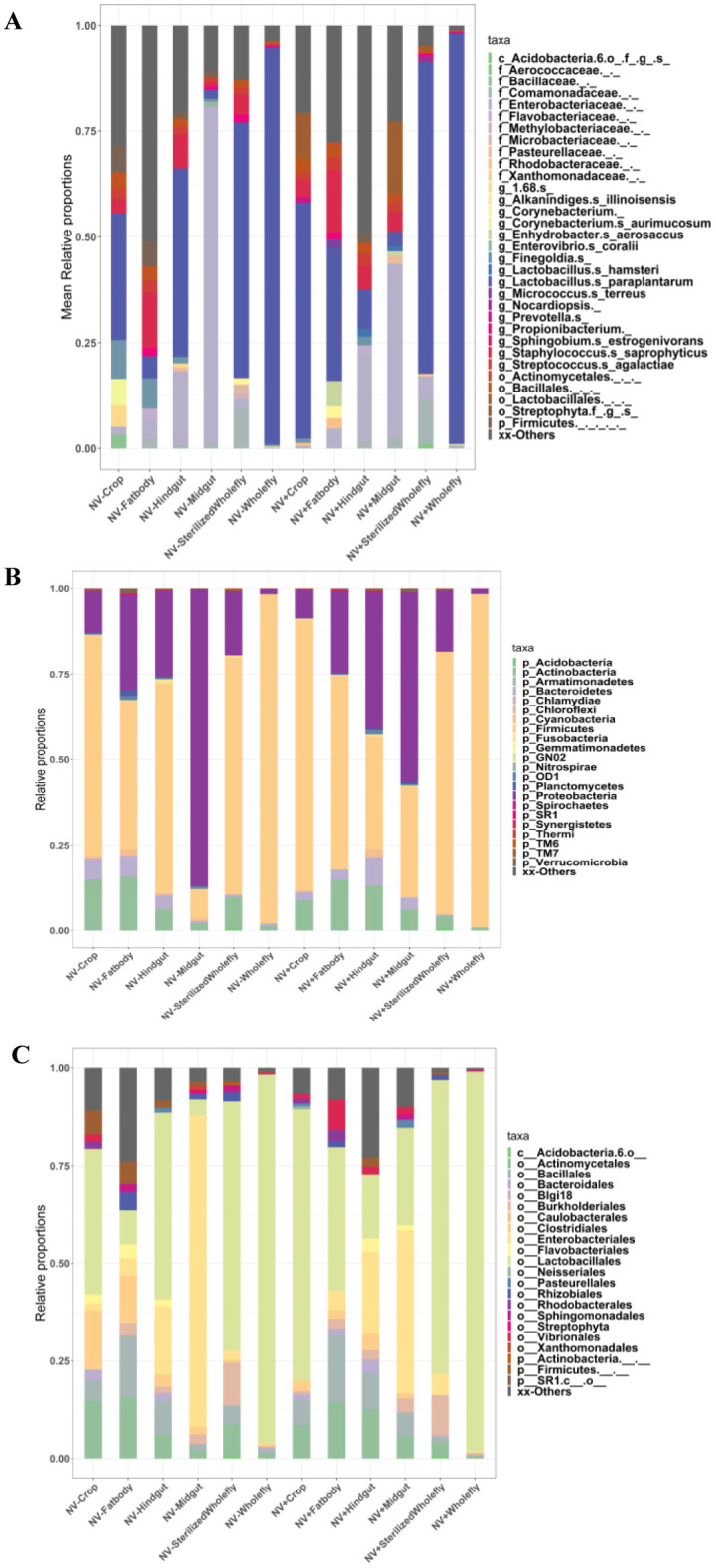
Relative abundance (from bacterial 16S rRNA gene sequences) of major phylogenetic groups at (A) phylum level, (B) order level and, (C) species level in different body sites of *D. melanogaster*. Negative Nora virus (NV-) infection: NV- Crop, NV- Fat body, NV- Midgut, NV- Hindgut, NV- Sterilized whole fly, and NV- Whole fly; Positive Nora virus (NV+) infection: NV+ Crop, NV+ Fat body, NV+ Midgut, NV+ Hindgut, NV+ Sterilized whole fly and NV+ Whole fly. (n = 3 replicates per treatment).

Bacteria species evenness was also assessed in this study. Results indicated that there is a significant difference in species evenness between the 5 body sites (p ≤ 0.05, Figure 5A). The fat body was the most even, while the whole fly was the least even, in terms of bacterial species. When the diversity within a community is even, the various bacteria species present have equal representation. There were also significant differences in bacteria species evenness between the sub-body sites, indicating that the NV- fat body was the least even and the sterilized NV+ whole fly was the most even (p ≤ 0.01, Figure 5B). The results demonstrated that there was also a significant difference in bacteria species evenness between the NV- hindgut and the NV+ hindgut (p ≤ 0.05, Figure 5D) and between the NV+ crop and the NV+ hindgut (Figure 5G). Finally, when comparing NV+ samples and NV− samples, there was no significant difference in bacteria species evenness (Figure 5C). Phylogenetic diversity analysis (Faith's PD) was continued to explore any differences in phylogenetic diversity between body sites and sub-body sites. There are significant differences between the 5 body sites, demonstrating that the bacteria in the crop are the least phylogenetically diverse and the sterilized whole fly and whole fly are the most diverse (p < 0.05, Figure 6A). There are also significant differences in phylogenetic diversity of the bacteria present in the different sub-body sites. The NV+ crop contained bacteria that were the least phylogenetically diverse, whereas the bacteria present in the NV+ sterilized whole fly are the most diverse (p ≤ 0.01, Figure 6B). Again, when comparing NV+ and NV− samples, there was no significant difference in bacteria species phylogenetic diversity (Figure 6C). Other significant differences of bacteria species phylogenetic diversity in sub-body sites include: NV− crop vs. NV− fat body, NV- fat body vs. NV− hindgut, NV-sterilized whole fly vs. NV-whole fly, NV+ crop vs. NV+ hindgut, NV− sterilized whole fly vs NV+ sterilized whole fly and NV+ crop and NV+ midgut (Figure 6D-I, p ≤ 0.05). Taxonomic analysis was performed and each sample showed the presence of a diverse range of bacterial species in each body site of *Drosophila*. The whole fly samples, regardless of Nora virus infection or not, had a large relative proportion of *Lactobacillus* species, whereas internal organs demonstrated a higher diversity of other bacterial species (Figure 7). We observe Firmicutes, Proteobacteria, Actinocteria, and Bacteroidetes are major phyla found in NV− and NV+ samples of *Drosophila* body sites. The major taxa at order level are from Lactobacillales, Actinomycetales, Bacteroidales, Clostridiales, Enterobacteriales, Flavobacteriales, Burkholderiales, Sphingomonadales and Bacillales.

## 4. Discussion and conclusion

This study was conducted to explore the potential symbiotic relationship between Nora virus and the innate microbiota found in *D. melanogaster*. Axenic stocks were created by using flies that had never been exposed to the antibiotics and allowed to lay eggs on the food for 4 days. They were transferred to new food bottles that contained the antibiotics, as the F1 emergents would contain microbiota susceptible to the antibiotics [39]. The flies were confirmed for Nora virus infection via RT-PCR (Figure 1) and by microbial analysis found to be free of bacteria. Four treatment groups, NV+/B+, NV-/B+, NV+/B−, NV−/B−, were used for longevity analysis. When comparing all four groups, flies that were NV−/B+ lived the longest, while those that were NV−/B− lived the shortest amount of time (Figure 2). The uninfected but colonized flies (NV−/B+) lived significantly longer (p ≤ 0.0001; 39 days), compared to the infected and colonized flies (NV+/B+) that only lived as long as 27 days. This suggests that bacterial colonization, to a much greater extent than Nora virus infection, is important to longevity. When comparing Nora virus infected groups with Nora virus uninfected groups, the data show that Nora virus may be slightly beneficial to the life span of *D. melanogaster*, if the fly is not colonized with bacteria (Figure 2; NV+/B− vs NV−/B−). Studies describing gastrointestinal Norovirus discovered that mice with persistent viral infection may have a longer life span than those without the persistent viral infection [21],[40]. Unlike other picorna and picorna-like viruses, Nora virus has only shown a slight impact on the life span of *D. melanogaster*. Nora virus infection only demonstrates a negative impact on longevity past 50 days of survival, making it inconclusive if mortality is due to viral infection or aging [26]. Another study reported that Nora virus infection does not significantly affect longevity, which may be due to genetic variation [27]. Drosophila C virus is another virus that was believed to be a part of the *Picornaviridae* family due to its size and protein similarities [41]. This virus has demonstrated extreme effects on the longevity of *D. melanogaster* with a mortality rate of 100% at 7 days post-viral inoculation [42]. Another picorna-like virus, Triatoma virus, found in *Triatoma infestans*, also has a negative impact on life span, increasing mortality rates to 60% [43]. In the future, it will be interesting to determine the genetics that underly the role Nora virus infection has on life span in *D. melanogaster*.

When comparing *D. melanogaster* with and without native gut bacteria, the data show that the bacteria are crucial in sustaining the life of the fly (Figure 2). While uninfected flies colonized with their native gut bacteria lived as long as 39 days, the uninfected flies receiving antibiotic treatment (not colonized) lived only 9 days (30 day difference; p ≤ 0.0001). Other studies using *Drosophila* have demonstrated conflicting results when testing longevity on axenic stocks. Numerous studies demonstrated that the reduction of gastrointestinal bacteria with use of antibiotics substantially increased longevity in *Drosophila* [44]–[49]. It has also been demonstrated in *Drosophila* that the use of antibiotics to create axenic stocks has no impact on longevity at all [50]. A final study demonstrated that when *D. melanogaster* is protected from bacteria during their first week of adulthood, as their lives are shortened by a third [7]. As shown in other organisms, such as mice, maintaining a healthy gastrointestinal tract via increased levels of gut microbes significantly lengthens the life of the organism [51]. Microbiota in *D. melanogaster* is very important as it promotes homeostasis in the gut and has a large impact on gut immunity [52]. The dramatic decrease in longevity in the axenic (NV−/B−) flies did not go unnoticed. *Drosophila* with different genetic backgrounds were found to have different microbial flora [53] and life span [54], which may contribute to the results seen. The Witi *Rel^E23^* strain used in this experiment was previously compared to Canton-S and found not to be significantly different in response to exposure to bacteria or life span [30]. Even so, we cannot discount that the genetic background of the strain used may contribute to the marked decrease in longevity and must be further studied. In addition, the short life span of NV−/B− flies may also be a result of the bleaching and antibiotic treatments. Lee *et al*. found a 10.16% decrease in mean life span of flies that had been exposed to 5% bleach treatment. In addition, they found that antibiotic dose appeared to have a toxic effect on axenic flies [55]. Even though our flies were created to be axenic following Brummel *et al*. [7], the combination of the genetic background and axenic treatment could have contributed to the decrease in life span. Therefore, characterizing the gut microbiota in different strains is important in understanding gut immunity in *D. melanogaster* as a whole.

The results from the longevity study led us to hypothesize that there may be differences in gastrointestinal bacterial communities between Nora virus infected and uninfected *D. melanogaster*. Virgin female flies were collected and aged for 4 days prior to dissection to allow bacterial communities from the larval stage to deplete before developing the adult microbiota [56]. The results from the metagenome analysis demonstrated that the DNA extractions were sufficient for analysis as indicated via the 16S sequence quality results (Supplementary Figure 1). The rarefaction curves constructed from Shannon diversity index for each sample tended to be flat with increasing sequencing quantity, indicating that the sequencing depth was sufficient to cover the microbial diversity in each sample (Figure 3). Nora virus uninfected flies contained fewer bacteria species than Nora virus infected flies. This is consistent in the literature, suggesting that bacterial communities grow in number when viruses are present in the system, explaining why secondary bacterial infections are so common during viral infection [57]–[59]. Diversity and richness of bacteria were analyzed for both NV+ and NV− whole body sites, as well as each sub-body site (Figure 3). These results indicate that the whole fly is the most diverse, while the crop show the most richness. It is expected that the entire body would be more diverse when compared to a single sub-body site. Due to the novelty of this study, there is little known about phylogenetic diversity and richness in various body sites compared to the whole system in *Drosophila*. However, this type of analysis has been used to assess the various microbial communities present at different instar stages of *Ceratitis capitata*, the Mediterranean fruit fly. The results from that study indicated that the pupal stage contained the greatest amount of phylogenetic diversity compared to the 1^st^ and 3^rd^ instar larva, as well as the adult fly [60]. Results from the sub-body sites conclude that the NV+ fat body is the most diverse, while the NV− fat body has the most richness (Figure 3). A study exploring the bacteria diversity in various gut sections of the *Panchlora* cockroach described that the fat body oftentimes contains the endosymbiont that is classified as *Blattabacterium*, which is not known to infect the rest of the gut of the organism. This endosymbiont is also present in many termite species. The researchers were unable to fully extract the fat body prior to metagenomic analysis, so all sequences that were associated with the endosymbiont were removed prior to analysis [61].

When bacterial phylogeny was compared among the different body sites, the results demonstrated that the bacteria present NV− crop and NV+ crop had very different bacteria from NV− fat body, NV− midgut, and the whole fly (p ≤ 0.05; Figure 4). This finding is not particularly surprising as the fat body and midgut are located in two separate areas of either the gut or the organism. Results from the evenness analysis demonstrated that there is a significant difference in bacteria species evenness between the 5 body sites (p ≤ 0.05, Figure 5A). Evenness is a depiction of species equality as a representation within a specific area. The results conclude that the NV- fat body was the least even and the sterilized NV+ whole fly was the most even (p ≤ 0.01, Figure 5B). These findings may allow for characterization of an endosymbiont present within the fat body of *D*. melanogaster that aids in Nora virus replication. Bacteria species evenness varied significantly between the NV− midgut and the NV− fat body (p ≤ 0.05, Figure 5E). Finally, when comparing NV+ samples and NV− samples, there was no significant difference in bacteria species evenness (Figure 5C). When exploring bacterial diversity within sub-body sites, the results indicated that the NV+ crop was the least phylogenetically diverse sub body site, while the NV+ whole fly was the most diverse (Figure 6). Early digestion within the gastrointestinal tract of *D. melanogaster* occurs within the crop (foregut) of the fly, but the exact role of this organ is still to be elucidated [62]. The gut is principally involved in excretion [63], whereas the fat body senses nutritional conditions, energy response and immune response [64]. Due to the differences in function and physiology between these three organs or sub-body types, it is not surprising that the bacterial microflora is different. Differences detected in the fat body are interesting, but unfortunately there are very few, if any microbiome studies that specifically address the fat body separately in *D. melanogaster*. Although extreme care was taken to not cross-contaminate organs and organ sections, it is something to consider. For example, the tracheal system is a network of branched tubules that extend throughout the body cavity and would be difficult to completely exclude from the dissected tissue. When dissections were being performed, extra care was taken to remove this material to get as clean of a dissection as possible. As we don't believe that this was an issue with our dissections, it is a possibility that cannot be discounted completely. Overall, this novel finding in the fat body suggests that perhaps Nora virus has an uncharacterized interaction here that needs to be further studied.

Of the bacteria present within the gastrointestinal tract of *D. melanogaster*, one of the most common genera is *Lactobacillus* [65]. *Lactobacillus* is a gram-positive, rod-shaped bacterium that serves a wide array of benefits to the gastrointestinal tract of *D. melanogaster*. In fact, it is possible that *Lactobacillus* alone has the ability to maintain a successful gastrointestinal system through growth regulation and hormone trafficking within *D. melanogaster* [8]. In addition, *Lactobacillus* bacteria are capable of restoring gut epithelium in *D. melanogaster*, which is especially important upon gastrointestinal infection [66]. In this study, we found that the major species in the whole-body preparations was *Lactobacillus* (Figure 7C). Along with *Lactobacillus* from the order Lactobacilliales, another common bacterial order normally found within the gastrointestinal tract of *D. melanogaster* is Enterobacteriales [65]. In the organs used in this study, both Lactobacilliales and Enterobacteriales, as well as numerous other orders, were found in different proportions. Of these, the most populous in the midgut were the Enterobacteriales, which was not found in as high of proportions in the whole fly (Figure 7C). This infers that these bacteria in this order are more specialized to the internal organs and not on the outside of the fly. These bacterial orders work together to benefit the organism in a variety of ways. They are found in the microflora of *D. melanogaster* and have the ability to influence mating preference, causing flies with similar microbiota make-up to mate with one another [13]. In addition, these bacteria are also capable of regulating gut immunity through the prevention of enteric viral infectivity. This works by antiviral pathways being upregulated in the midgut, which is where the majority of gut microbiota is found [18]. This has been demonstrated with human immunodeficiency virus infection that was shown to alter microbiota composition all over the body, specifically within the gastrointestinal tract [67]. In addition, when poliovirus is inoculated into mice, data show that the virus replicates at greater levels when the gut bacteria is present as opposed to antibiotic treatment [68]. This study was especially interesting because like Nora virus, poliovirus is in the *Picornaviridae* family.

The results from this experiment supported the benefits of gut microbiota in *Drosophila*, however, results were not conclusively in support of the hypothesis regarding the mutualistic relationship between Nora virus and the microbiota. There was a decrease in life span when flies colonized with bacteria were infected with Nora virus (NV−/B+ versus NV+/B+), but an increase in life span when bacteria were not present in infected flies (NV+/B− versus NV−/B−), which needs to be further investigated. Contrary to these results, a study conducted by Kernbauer *et al*. suggested there was a significant connection between virus and commensal bacteria found in the guts of mice [40]. This study also explored the diversity, richness, and evenness of bacterial species present between different sections of the gastrointestinal tract and fat body in Nora virus infected and uninfected *D. melanogaster*. The results consistently reported that the NV+ fat body contains the most bacteria, and that bacteria had the greatest amount of diversity present between all body sites. This might suggest that the fat body has an as of yet uncharacterized interaction with Nora virus and needs to be further investigated. This study is beneficial to the *Drosophila* community as it provides insight into longevity regarding bacterial and viral interaction, which is largely unaddressed.
