# Supplementary material for: Effect of Nora virus infection on native gut bacterial communities of Drosophila melanogaster
Source: AIMS Microbiol. 2021 Jun 10;7(2):216–37. doi: 10.3934/microbiol.2021014 (PMC8255909; doi:10.3934/microbiol.2021014)
Supplement: Supplementary file 1 [file microbiol-07-02-014-s001.pdf]

AIMS Microbiology

http://www.aimspress.com/journal/microbiology

AIMS Microbiology, 7(2): 216–237.
DOI: 10.3934/microbiol.2021014
Received: 04 February 2021
Accepted: 07 June 2021
Published: 10 June 2021

Research article

Effect of Nora virus infection on native gut bacterial communities of *Drosophila melanogaster*

Makayla Schissel [1], Rebecca Best [1], Shelby Liesemeyer [1], Yuan-De Tan [2], Darby J. Carlson [1], Julie J. Shaffer [1], Nagavardhini Avuthu [2], Chittibabu Guda [2], and Kimberly A. Carlson [1,*]

[1] Biology Department, University of Nebraska at Kearney, 2401 11th Ave, Kearney, NE 68849, USA
[2] Department of Genetics, Cell Biology and Anatomy, University of Nebraska Medical Center, 985805 Nebraska Medical Center, Omaha, NE 68198-5805, USA

* **Correspondence:** Email: carlsonka1@unk.edu; Tel: +13088651554; Fax: +13088658045.

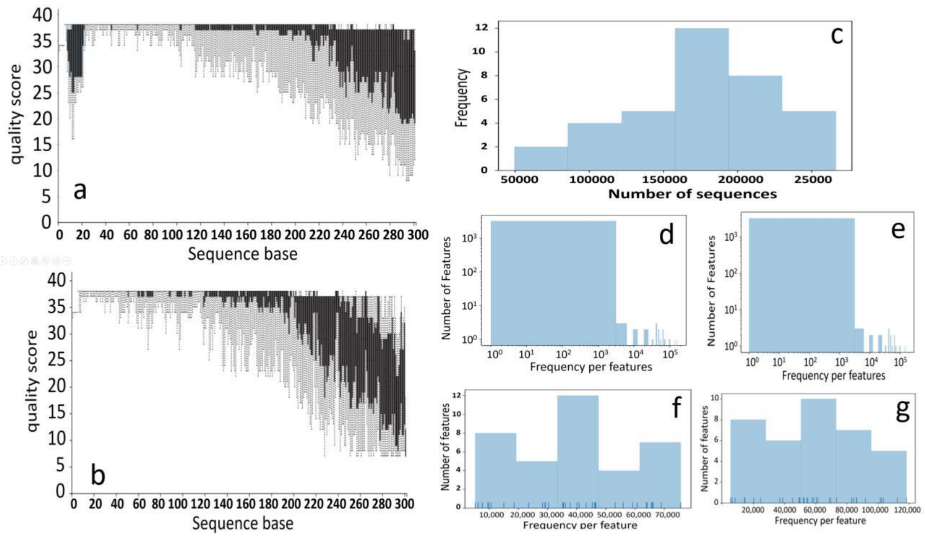

Supplementary Figure 1. 16S-DNA sequence quality (a) Interactive quality plot for forward of reads. (b) Interactive quality plot for reversion of reads. Forward and reversion read qualities were given by moving mouse prompt on the largest quality score of middle of the box. (c) Demultiplexed sequence counts summary. (d) Frequency of feature from table summary by dada2 method. (e) Frequency of feature from table summary by deblur method. (f) Frequency per sample from table summary by dada2 pipeline. (g) Frequency per samples from table summary by deblur method.

AIMS Press

© 2021 the Author(s), licensee AIMS Press. This is an open access article distributed under the terms of the Creative Commons Attribution License (http://creativecommons.org/licenses/by/4.0)
